# Clinical Performance of BAL Metagenomic Next-Generation Sequence and Serum (1,3)-β-D-Glucan for Differential Diagnosis of *Pneumocystis jirovecii* Pneumonia and *Pneumocystis jirovecii* Colonisation

**DOI:** 10.3389/fcimb.2021.784236

**Published:** 2021-12-22

**Authors:** Li Liu, Mingjuan Yuan, Yi Shi, Xin Su

**Affiliations:** ^1^ Department of Infectious Disease, Hunan Provincial People’s Hospital (The First Affiliated Hospital of Hunan Normal University), Changsha, China; ^2^ Department of Respiratory and Critical Care Medicine, Jinling Hospital, The First School of Clinical Medicine, Southern Medical University, Nanjing, China; ^3^ Department of Infectious Disease, The Central Hospital of Yueyang, Yueyang, China; ^4^ Department of Respiratory and Critical Care Medicine, Jinling Hospital, Medical School of Nanjing University, Nanjing, China

**Keywords:** mNGS, 1,3-β-D-glucan, *Pneumocystis jirovecii*, colonisation, PJP

## Abstract

**Background:**

Differentiating *Pneumocystis jirovecii* infection from colonisation is crucial for appropriate therapy administration. In this study, we evaluated the performance of bronchoalveolar lavage fluid (BAL) metagenomic next-generation sequencing (mNGS) and serum 1,3-β-D-glucan (BDG) tests in differentiating colonisation and infection with *P. jirovecii.*

**Methods:**

From January 2018 to March 2021, 47 patients were enrolled in this study at the Hunan Provincial People’s Hospital. The final diagnosis was used as a reference, and cases were classified into the *P. jirovecii* pneumonia (PJP) group or the *P. jirovecii* colonisation (PJC) group. Clinical data were recorded. The performances of mNGS and BDG were compared.

**Result:**

The fungal load significantly differed between patients with PJP and PJC, with median reads of 3,215.79 ± 1,797 *vs*. 5.61 ± 0.88 in the PJP and PJC groups, respectively (*P* < 0.0001). BDG also significantly differed between the two groups, with a median titre of 233.60 ± 39.65 pg/ml in the PJP group and 68.48 ± 19.21 pg/ml in the PJC group (*P =* 0.0006). The area under the curve was 0.973 (95%CI: 0.868–1.007) for mNGS of the BAL and 0.879 (95%CI: 0.769–0.989) for the serum BDG. The optimal threshold value for discriminating *P. jirovecii* infection from colonisation appeared to be 14 reads (sensitivity, 83.3%; specificity, 95.7%; positive likelihood ratio, 19.2) and BDG = 88.6 pg/ml (sensitivity, 79.2%; specificity, 92.9%; positive likelihood ratio, 18.2). No correlation between mNGS reads and the BDG titre was found in mNGS-positive patients (*r* ^2^ = 0.0076, *P* = 0.583). The levels of lactate dehydrogenase and C-reactive protein were significantly higher in the PJP group than in the PJC group.

**Conclusion:**

BAL mNGS and serum BDG are useful adjunct tests that can assist with differentiating between colonisation and infection of *P. jirovecii*.

## 1 Introduction

*Pneumocystis jirovecii* pneumonia (PJP) is a great threat to immunosuppressed patients (Liu et al., 2019), particularly in non-human immunodeficiency virus (HIV)-infected patients. However, *P. jirovecii* colonisation is a less severe presentation of *P. jirovecii* infection. In recent years, asymptomatic *P. jirovecii* colonisation has been detected in immunocompetent patients (Morris and Norris, 2012; Ma et al., 2018). Therefore, discriminating between these two clinical manifestations of *P. jirovecii* is critical.

The gold standard for diagnosing PJP is direct visualisation of pneumocysts using immunofluorescence microscopy (Kovacs and Masur, 2009). However, immunofluorescence staining of *P. jirovecii* is not routinely performed in many hospitals and shows low diagnostic sensitivity in patients (Limper et al., 1989). The molecular detection of *Pneumocystis* in bronchoalveolar lavage (BAL) has become an important diagnostic tool (Lu et al., 2011; Tia et al., 2012; Fan et al., 2013).

With the rapid development of molecular biology, metagenomic next-generation sequencing (mNGS) tests of respiratory samples are used to directly detect target organisms (Gu et al., 2019). Jiang et al. (2021) found that mNGS had a remarkably higher sensitivity of 100% compared to Gomori methenamine silver staining (25.0%) and serum (1,3)-β-D-glucan (BDG) (67.4%) for diagnosing PJP. However, differentiating between colonisation and infection by mNGS remains challenging. A positive result can be difficult to interpret, as it may reflect colonisation rather than infection. The absence of a reliable gold standard for diagnosing PJP is a key factor limiting the interpretation and application of mNGS.

According to consistent clinical manifestations, serum BDG can be used as an early screening tool to diagnose and initiate treatment for PJP. Various meta-analyses of clinical evaluations have focused on the performance of the BDG assay for diagnosing PJP (Karageorgopoulos et al., 2013; Del Corpo et al., 2020). Most studies of the serum BDG test in PJP focused on patients with HIV and showed that the overall sensitivity of this test for the diagnosis of PJP was 91%; negative results were excluded (Karageorgopoulos et al., 2013). A recent study of the clinical performance of BDG in patients with cancer in conjunction with qualitative real-time P. *jirovecii* PCR in the BAL (Morjaria et al., 2019) showed that an elevated BDG threshold (>200 pg/ml) can help distinguish possible *P. jirovecii* colonisation from active infection in *P. jirovecii* RT-PCR-positive patients.

Currently, mNGS is broadly applied to detect organisms in BAL fluid. No studies have evaluated the diagnostic potential of combining serum BDG and BAL mNGS for differentiating between colonisation and infection of *P. jirovecii.* In this study, we analysed the performance of BAL mNGS and serum BDG to discriminate PJP infection from colonisation.

## 2 Methods

### 2.1 Study Design and Participants

This retrospective study was conducted at Hunan Provincial People’s Hospital (Hunan, China) between January 2018 and March 2021. Patients with unexplained lung infiltrates and clinical suspicion of PJP who underwent BAL mNGS testing during the study period were enrolled. The highest level of serum BDG detected within a week of mNGS was used for analysis. Patients were excluded if the following criteria were met: (1) administered empiric treatment (therapeutic doses) for PJP prior to the mNGS assay, (2) underwent the serum BDG assay more than 7 days from the mNGS, and (3) had other pulmonary fungal infections. Before the end of the study, we collected detailed data on patient demographics, type of malignant tumour, haematological malignancies, acquired immunodeficiency syndrome (AIDS) and HIV infection status, use of corticosteroids within the previous 90 days, and autoimmune disease. Additionally, inflammatory markers, such as white blood cell count, procalcitonin, C-reactive protein (CRP), and lactate dehydrogenase (LDH), within 7 days before mNGS were collected. Two independent clinicians reviewed all cases to determine the final diagnosis of PJP. The final diagnosis was used as a reference to which the mNGS results were compared. Patients with a positive mNGS result but without a final diagnosis of PJP were considered as colonised. If the result of mNGS was negative, the patient was considered as not infected by *P. jirovecii.* This retrospective study was approved by the Ethics Committee of Hunan Provincial People’s Hospital.

### 2.2 Definitions

PJP infections were defined as either definite/probable, possible, or not PJP, as described for other invasive fungal diseases, with some modifications (De Pauw et al., 2008).

Definite/probable PJP was defined as cases associated with positive microscopy/positive mNGS in the BAL sample together with at least two of the following four clinical manifestations—dyspnoea, fever, dry cough, or hypoxemia (O_2_ saturation <90% on room air)—and classical radiological signs (both-sided opacification on chest computed tomography, widespread pulmonary infiltrates). Possible PJP was defined as patients showing negative microscopy results but with a positive result in BAL mNGS with no symptoms or radiological signs of PJP.

Non-PJP controls included patients who showed negative microscopy and mNGS results in the BAL and had no symptoms or radiological signs of PJP.

### 2.3 Procedure

#### 2.3.1 Serum BDG Test

Serum BDG was detected using the Fungitell assay according to the manufacturer’s instructions (Dynamiker Biotechnology, Tianjin, China). The result was considered as positive when the BDG value was ≥80 pg/ml, according to the manufacturer’s instructions. No further dilutions were performed when the BDG result was ≥600 pg/ml.

#### 2.3.2 BAL mNGS

The BAL fluid was collected by experienced bronchoscopists after anaesthesia with midazolam in accordance with standard procedures at Hunan Provincial People’s Hospital. Briefly, the sampling location was selected according to chest computed tomography images. Sterile saline (20 ml) was instilled into the target subsegmental bronchi three times. The BAL fluid was retrieved gently by syringe suction, and the first 20 ml was typically discharged to avoid contamination, whereas the remaining samples were placed in sterile containers and immediately sent to the BGI-Huada Genomics Institute (Wuhan, China) for the mNGS test.

The BAL fluid (2 to 3 ml) was mixed and shaken with glass beads, then attached to a horizontal platform on a vortex mixer, and agitated vigorously at 2,800–3,200 rpm for 30 min. DNA was extracted using the TIANamp MicroDNA kit (Tiangen Biotech, Tiangen, China) according to standard procedures. The extracted DNA was ultrasonically broken into 200–300-base pair fragments. An Agilent 2100 Bioanalyzer (Agilent Technologies, Santa Clara, CA, USA) was used for quality control of the library, and a Qubit dsDNA HS Assay Kit (Thermo Fisher Scientific, Waltham, MA, USA) was used for quality control of the DNA library concentration. To control for contamination in sequencing, we added a negative control to each run. When the whole process showed good performance, we duplicated one sample in each run for monitoring. If the replicates produced the same results, the results were considered as reliable.

Low-quality short reads (length <35 base pairs) were removed. High-quality sequencing data were generated by computational subtraction of human host sequences mapped to the human reference genome (hg19) using Burrows-Wheeler Alignment (0.7.10-r789). The remaining sequences were aligned to the current virus, bacteria, fungi, and protozoa databases from NCBI (https://www.ncbi.nlm.nih.gov/genomes), which are comprised of whole-genome sequences of 4,061 viral taxa, 2,473 bacterial genomes or scaffolds, and genomic sequences for 199 fungi related to human infection and 135 parasites associated with human diseases.

### 2.4 Statistical Analysis

The sensitivity and specificity for the diagnosis of *P. jirovecii* pneumonia were determined for serum BDG and BAL mNGS. Receiver operating characteristic curves were drawn, and cut-off values were selected to assess the diagnostic accuracy of serum BDG and BAL mNGS for PJP *versus P. jirovecii* colonisation (PJC) groups. Data analyses were performed using SPSS software (version 20.0; SPSS, Inc., Chicago, IL, USA). The figures were constructed using GraphPad Prism 5 software (GraphPad, Inc., La Jolla, CA, USA). Statistical significance was set at a *P*-value less than 0.05.

## 3 Results

### 3.1 Population Characteristics

**Chart 1:**
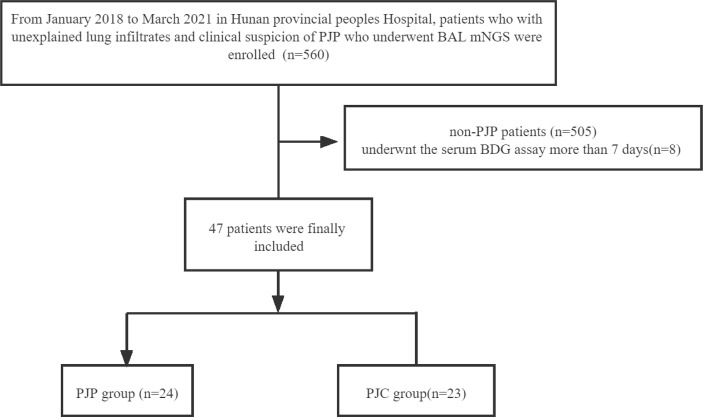
Flow diagram of the patients included in the study.

Over a 39-month study period, respiratory samples were obtained through BAL (*n* = 560) and subjected to mNGS; 55 patients showed a positive result in the *P. jirovecii* mNGS test and were categorised into the definite/probable PJP group (*n* = 29), possible PJP group (*n* = 26), and non-PJP group (*n* = 505). Serum BDG testing was performed within 7 days of mNGS in 47 patients, and the final diagnosis was used as the reference; these cases were classified into the PJP group (*n* = 24) and the PJC group (*n* = 23) (**Chart 1**). The mean age of the PJP group was 55 ± 2.37 years, and the host factors included AIDS (*n* = 2), solid tumour with chemotherapy within 3 months (*n* = 7), haematological malignancies (non-Hodgkin’s lymphoma, Hodgkin’s lymphoma, lymphoproliferative diseases) (*n* = 6), chronic renal disease (*n* = 5), autoimmune disease (*n* = 2), and others (*n* = 2). The PJC group consisted of 19 men and 4 women, with a mean age of 58 ± 3.02 years. The host factors included solid tumours with chemotherapy (*n* = 7), chronic pulmonary disease (*n* = 7), chronic renal disease (*n* = 2), autoimmune disease (*n* = 3), and others (*n* = 4). Corticosteroid use, fever, and both-sided opacification on chest computed tomography were more common in the PJP group than in the PJC group (**Table 1**).

**Table 1:** Demographics and clinical characteristics of *Pneumocystis jirovecii* pneumonia (PJP) and *Pneumocystis jirovecii* colonisation (PJC) patients.

Characteristic	PJP	PJC	*P*-value
Patients	*24*	23	
Age, median (years)	55 ± 2.37	58 ± 3.02	
Female/male	6/18	4/19	
Underlying condition, number of patients			
Solid tumour	7/24	7/23	
Hematologic malignancy	6/24	0/23	
Chronic renal disease	5/24	2/23	
Autoimmune disease	2/24	3/24	
HIV	2/24	0/23	
Chronic pulmonary disease	*0/24*	7/23	
Other	2/24	4/23	
Corticosteroid use	13/24	1/23	*P* = 0.0003
Clinical presentation			
Fever	17/24	6/23	0.003
Dry cough	15/24	15/23	1.0
Dyspnoea	19/24	12/23	0.068
Hypoxemia	15/24	13/23	0.110
Both-sided opacification on chest CT	18/24	0/23	<0.0001
Laboratory test			
WBC (×109/L; normal range:3.5–10)	9.68 ± 1.19	7.76 ± 0.91	*P* = 0.208
PCT (ng/ml; normal range: 0–0.5)	1.71 ± 0.76	3.65 ± 3.15	*P* = 0.547
CRP (mg/L; normal range: 0–5)	99.33 ± 19.37	46.92 ± 11.80	*P* = 0.027
LDH (U/L; normal range: 100–240)	472.9 ± 38.99	302.8 ± 30.84	*P* = 0.001

WBC, white blood cell; PCT, procalcitonin; CRP, C-reactive protein; LDH, lactate dehydrogenase; CT, computed tomography.

**Figure 1:**
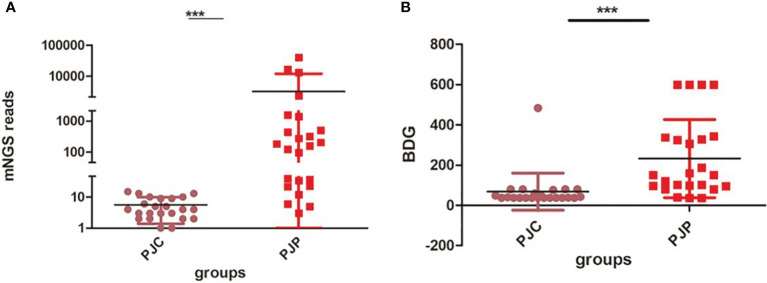
Test of metagenomic next-generation sequencing (mNGS) reads and 1,3-β-D-glucan (BDG) between the *Pneumocystis jirovecii* pneumonia (PJP) and *Pneumocystis jirovecii* colonisation (PJC) groups. **(A)** Test of mNGS reads between the PJP and PJC groups. **(B)** Test of BDG titre between the PJP and PJC groups ***P < 0.0001.

**Figure 2:**
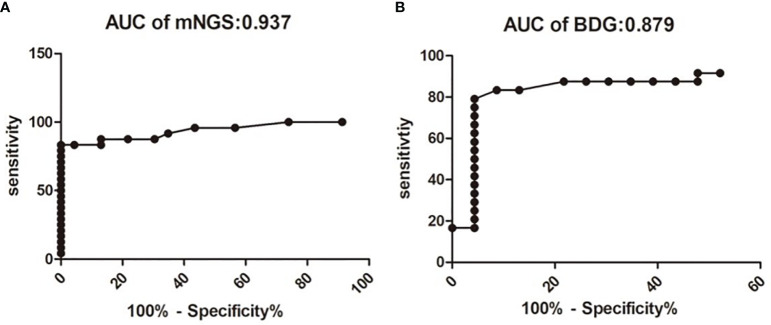
Receiver operating characteristic curve for metagenomic next-generation sequencing (mNGS) and serum b-D-glucan assay for discrimination between *Pneumocystis jirovecii* pneumonia and *Pneumocystis* colonisation. The area under the curve value of mNGS is 0.937 **(A)**. The area under the curve value of mNGS is 0.879 **(B)**.

The fungal load was significantly higher in the PJP group, with a median mNGS read number of 3,215.79 ± 1,797 *vs*. 5.61 ± 0.88 in the PJC group (*P* < 0.0001, **Figure 1A**). BDG was also significantly higher in the PJP group, with a median titre of 233.60 ± 39.65 *vs*. 68.48 ± 19.21 pg/ml in the PJC group (*p =* 0.0006, **Figure 1B**). The area under the curve was 0.973 (95%CI: 0.868–1.007) for mNGS in BAL and 0.879 (95%CI: 0.769–0.989) for serum BDG. The optimal threshold values for discriminating *P. jirovecii* infection from colonisation appeared to be 14 reads (sensitivity, 83.3%; specificity, 95.7%; positive likelihood ratio, 19.2; **Figure 2A**) and BDG of 88.6 pg/ml (sensitivity, 79.2%; specificity, 92.9%; positive likelihood ratio, 18.2; **Figure 2B**).

**Figure 3:**
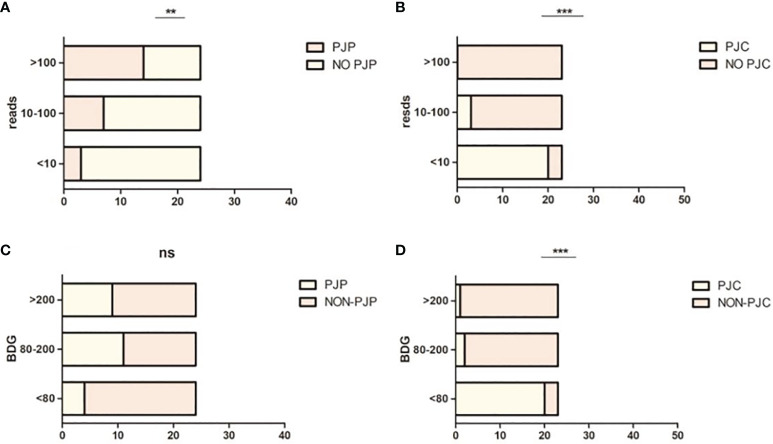
Proportion of *Pneumocystis jirovecii* pneumonia (PJP) and *Pneumocystis jirovecii* colonisation (PJC) in different metagenomic next-generation sequencing (mNGS) reads and 1,3-β-D-glucan (BDG) titre. **(A)** Proportion of PJP in different mNGS reads. **(B)** Proportion of PJC in different mNGS reads. **(C)** Proportion of PJP in different BDG titre. **(D)** Proportion of PJC in different BDG titre. **P = 0.003; ***P < 0.0008; ns P = 0.0874.

When mNGS was divided into low (≤10), median (10–100), and high (≥100) read counts, the proportions of patients with PJP were 12.5% (3/24), 29.2% (7/24), and 58.3% (14/24), respectively (*χ* ^2^ = 11.63, *P* < 0.003; **Figure 3A**). When mNGS was divided into low (≤10), median (10–100), and high (≥100) read counts, the proportions of patients with PJC were 87% (20/23), 13% (3/23), and 0% (0/23), respectively (*χ* ^2^ = 45.52, *P* < 0.0001; **Figure 3B**).

**Figure 4:**
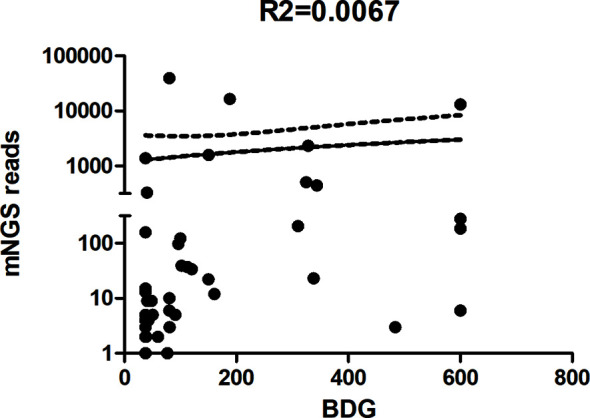
Scatterplot between reads of metagenomic next-generation sequencing (mNGS) and the 1,3-β-D-glucan titre in mNGS-positive patients.

When BDG was divided into low (≤80 pg/ml), median (80–200 pg/ml), and high (≥200 pg/ml), the proportions of patients with PJP were 16.7% (4/24), 45.8% (11/24), and 37.5% (9/24), respectively (*χ* ^2^ = 4.875, *P* < 0.0874; **Figure 3C**). When BDG was divided into low (≤80 pg/ml), median (80–200 pg/ml), and high (≥200 pg/ml), the proportions of patients with PJC were 87% (20/23), 8.7% (2/23), and 4.3% (1/23), respectively (*χ* ^2^ = 44.74, *P* < 0.0001; **Figure 3D**). The scatterplot showed no correlation between the mNGS reads and BDG titre in mNGS-positive patients (*r* ^2^ = 0.0076, *P* = 0.583; **Figure 4**).

We evaluated the performance of combining a threshold of 88.6 pg/ml of BDG and 14 reads of mNGS for diagnosing PJP. These criteria showed a sensitivity of 90% (95%CI: 68.3–98.8%), specificity of 25% (95%CI: 0.6–80.6%), positive predictive value of 85.7% (95%CI: 63.7–90.7%), and negative predictive value of 33.3% (95%CI: 0.8–90.6%; **Table 2**). We also evaluated the performance when BDG was less than 88.6 pg/ml and mNGS <14 reads for diagnosing PJC, which showed a sensitivity of 95.5% (95%CI: 77.2–99.9%), specificity of 0% (95%CI: 0–97.5%), positive predictive value of 95.5% (95%CI: 77.2–99.9%), and negative predictive value of 0% (95%CI: 0–97.5%; **Table 3**).

**Table 2:** Performance of combined beta-D glucan and mNGS in all PJP patients.

	PJP mNGS ≥14 reads	PJP mNGS <14 reads
BDG (≥88.6 pg/ml)	18	3
BDG (<88.6 pg/ml)	2	1
Total	20	4
Sensitivity	90%	95%CI (68.3–98.8%)
Specificity	25%	95%CI (0.6–80.6%)
PPV	85.7%	95%CI (63.7–90.6%)
NPV	33.3%	95%CI (0.8–90.6%)

BDG, beta-D glucan; mNGS, metagenomic next-generation sequence; PJP, Pneumocystis jirovecii pneumonia; CI, confidence interval; NPV, negative predictive value; PPV, positive predictive value.

**Table 3:** Performance of combined beta-D glucan and mNGS in all PJC patients.

	PCP mNGS <14 reads	PCP mNGS ≥14 reads
BDG (<88.6 pg/ml)	21	1
BDG (≥88.6 pg/ml)	1	0
Total	22	1
Sensitivity	95.5%	95%CI (77.2–99.9%)
Specificity	0	95%CI (0–97.5%)
PPV	95.5%	95%CI (77.2–99.9%)
NPV	0	95%CI (0–97.5%)

BDG, beta-D glucan; mNGS, metagenomic next-generation sequence; PCP, Pneumocystis jirovecii colonization; CI, confidence interval; NPV, negative predictive value; PPV, positive predictive value.

**Figure 5:**
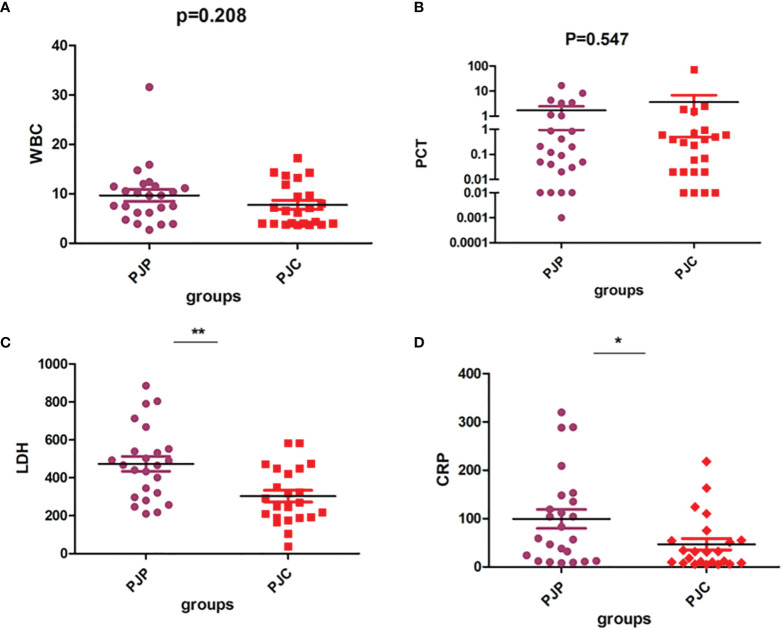
Test of white blood cell (WBC), procalcitonin (PCT), lactate dehydrogenase (LDH), and C-reactive protein (CRP) between the *Pneumocystis jirovecii* pneumonia (PJP) and *Pneumocystis jirovecii* colonisation (PJC) groups. **(A)** Test of WBC between the PJP and PJC groups. **(B)** Test of PCT between the PJP and PJC groups. **(C)** Test of LDH between the PJP and PJC groups. **(D)** Test of CRP between the PJP and PJC groups. **P = 0.001; *P = 0.027.

We observed no significant difference between patients with PJP and PJC in terms of white blood cell count and procalcitonin levels (**Figures 5A, B** and **Table 1**). However, the levels of LDH and CRP were significantly higher in the PJP group than in the PJC group (**Figures 5C, D** and **Table 1**).

## 4 Discussion

Timely and accurate diagnosis is the most important factor for improving PJP-related mortality in non-HIV-infected individuals (Del Corpo et al., 2020). The definitive diagnosis of PJP remains challenging, primarily because the organism cannot be cultured in clinical laboratories (Kovacs et al., 2001). The clinical importance of a positive *Pneumocystis* mNGS result has been questioned, particularly when low reads of *Pneumocystis* are detected. Therefore, mNGS requires standardisation. mNGS is a highly sensitive method for detecting *Pneumocystis* (Chen et al., 2020; Jiang et al., 2021; Zhang et al., 2021). However, it is difficult to differentiate between colonisation and true infections. Therefore, determining the mNGS read threshold can provide physicians with accurate information. Based on a careful review of clinical features, the cut-off value can be used to discontinue or initiate anti-PCP therapy. To date, no studies have assessed the performance of mNGS cut-off values for distinguishing PJP from colonisation. In this study, we retrospectively enrolled patients with a positive *P. jirovecii* mNGS test between January 2018 and March 2021 and evaluated the performance of BAL mNGS and serum BDG in assisting in the diagnosis of PJP.

Our analysis revealed the excellent ability of mNGS to discriminate PJP from colonisation with a suggested cut-off of 14 reads. mNGS showed excellent diagnostic performance for PJP. However, four patients with PJP showed read counts of fewer than 14 (3, 5, 6, and 12 reads, respectively); thus, the mNGS result must be carefully interpreted considering the clinical findings and radiological signs when mNGS reads are fewer than 14.

Immunofluorescence staining of *P. jirovecii* is the gold standard for diagnosing PJP. We compared the time required for pathogen identification using mNGS and immunofluorescence staining. The median turnaround time for obtaining mNGS results from BAL submission to pathogen identification was 2.5 days, which is longer than immunofluorescence staining. Critical value reports using immunofluorescence staining were obtained within 24 h, and this method requires less equipment, technical expertise, and cost compared to mNGS. However, immunofluorescence shows low positivity, and immunofluorescence staining of *P. jirovecii* is not routinely performed in many hospitals.

The proportion of mixed pulmonary infections in patients with PJP was much higher than originally predicted (Jiang et al., 2021). The main reason for this is incomplete immunity. Immunosuppressive therapies are the main cause of low immunity, and PJP occurs in non-HIV individuals (Rasche et al., 2016; De Simone et al., 2017). In this study, mNGS detected 10 patients with mixed infections by multiple pathogens. We did not evaluate these patients in detail, as this study focused on differentiating between colonisation and infection of *P. jirovecii.*

Although mNGS has many advantages for detecting pathogens, several challenges may hinder its application in the clinical setting. First, samples may be easily contaminated by environmental microbes or human parasitic bacteria during sampling or sequencing, making it difficult to determine which pathogen is involved in colonisation, infection, or contamination (Simner et al., 2018). Currently, no authoritative guideline is available for interpreting mNGS results, making interpretation of the results difficult for clinicians (Simner et al., 2018; Dulanto Chiang and Dekker, 2020). Therefore, effective and unified standards must be established to interpret the results. The high cost of mNGS also limits its clinical application. The cost-effectiveness of the broad application of mNGS requires further investigation (Thakur, 2020).

The European Conference on Infections in Leukaemia guidelines suggest that serum BDG can help diagnose PJP (Maertens et al., 2016). Although the sensitivity of the BDG test is high, a false-positive result is likely in the presence of factors such as haemodialysis, Gram-negative bacteraemia, severe mucositis, or use of intravenous immunoglobulins and some antibiotics (Onishi et al., 2012; Karageorgopoulos et al., 2013; Li et al., 2015; Azoulay et al., 2016). In addition, BDG cannot differentiate from a broad range of fungal pathogens. BDG levels alone cannot confirm the existence of PJP, and the results must be interpreted in combination with clinical findings. However, with a negative predictive value of up to 96%, a BDG level of less than 80 pg/ml almost rules out PJP (Alanio et al., 2016). Our findings are consistent with those of previous studies and indicate that the BDG assay is useful for ruling out PJP. The optimal threshold BDG values for discriminating between PJP and PJC appear to be 88.6 pg/ml, with a sensitivity of 83.3% and specificity of 95.7%. This is consistent with the findings of (Morjaria et al., 2019) who suggested that a serum BDG level of <80 pg/ml has a high negative predictive value (95.2%) which was derived from more than 400 patients with lower airway disease who underwent evaluations for PCP. This is also consistent with other studies primarily performed in patients (Held et al., 2011; Sax et al., 2011).

Some authors reported that the burden of *P. jirovecii* organisms in the lungs is typically heavier in patients with HIV and PJP than in non-HIV PJP patients (Salerno et al., 2014). Previous studies suggested that serum BDG can be used to evaluate the fungal burden. However, Held et al. (2011) showed that BDG did not correlate with the *P. jirovecii* burden. Thus, we also analysed the correlation between the mNGS reads and BDG titre in mNGS-positive patients. In agreement with the results of Held et al., no correlation was found between mNGS reads and BDG titre in mNGS-positive patients in our study.

When we used the threshold of 88.6 pg/ml BDG for conjunction with 14 reads of mNGS for diagnosing PJP, the sensitivity was excellent (90%), strongly supporting a diagnosis of PJP. The positive predictive value was up to 95.5% when combined with a BDG of less than 88.6 pg/ml and mNGS <14 reads for diagnosing PJC. Our results indicate that serum BDG combined with BAL mNGS showed excellent performance for differentiating between colonisation and infection of *P. jirovecii.*

Previous studies showed that more than 90% of patients with PJP have increased levels of biochemical indicators, such as CRP, erythrocyte sedimentation rate, LDH, and β-glucan (García-Lorda et al., 2000; Mercier et al., 2019). Thus, we evaluated inflammatory markers such as white blood cell count and procalcitonin, CRP, and LDH values between the two groups. In accordance with the findings of previous studies, we found that the levels of LDH and CRP were significantly higher in the PJP group than in the PJC group. However, LDH and CRP showed a very limited value in predicting PJP, which is consistent with previous studies demonstrating that they had high sensitivity for PJP but had limited specificity. These molecules are also increased in other lung infections in addition to PJP (García-Lorda et al., 2000).

Few studies have focused on the transition from colonisation to active disease in *P. jirovecii*. Detecting an infection early is important for preventing the colonisation of *P. jirovecii*, which will be evaluated in our further studies of *P. jirovecii* pneumonia.

Our study has some limitations. First, this was a retrospective study, and thus the researcher did not control for limited data and data accumulation. Second, we evaluated a relatively small sample size, and larger studies are needed to confirm our results. This was not a randomised controlled study and was performed in a single centre, limiting the generalisability of the results. A standard comparator for diagnosis was lacking, and classification bias may have occurred. Finally, the mNGS data in this study only provided reads from the mNGS service. No genome coverage was provided, and we only discussed *P. jirovecii* and did not report other organisms in detail.

In conclusion, the combined BALF mNGS results and serum BDG could help distinguish infection from colonisation of *P. jirovecii* and may be useful for guiding therapeutic decisions.

## Data Availability Statement

The datasets presented in this study can be found in online repositories. The names of the repository/repositories and accession number(s) can be found below: https://db.cngb.org/, CNP0002293.

## Ethics Statement

All procedures performed in this study involving human participants were conducted in accordance with the ethical standards of the Ethics Committee of the Hunan Provincial People’s Hospital, the 1964 Helsinki declaration and its later amendments, and with comparable ethical standards.

## Author Contributions

LL and MY collected the original clinical data and processed the statistical data. LL drafted and edited the manuscript. YS participated in the design, and XS revised the manuscript. All authors contributed to the article and approved the submitted version.

## Funding

This work was supported by the Natural Science Foundation of China project (grant no. 82070011), the Key Project of Jiangsu Commission of Health (grant no. K2019004), and the Changsha Municipal Natural Science Foundation (grant no. Kq2014193).

## Conflict of Interest

The authors declare that the research was conducted in the absence of any commercial or financial relationships that could be construed as a potential conflict of interest.

## Publisher’s Note

All claims expressed in this article are solely those of the authors and do not necessarily represent those of their affiliated organizations, or those of the publisher, the editors and the reviewers. Any product that may be evaluated in this article, or claim that may be made by its manufacturer, is not guaranteed or endorsed by the publisher.
